# The influence of extraprostatic extension grade on the detection of pelvic lymph node metastasis in prostate cancer

**DOI:** 10.3389/fonc.2025.1663809

**Published:** 2025-10-22

**Authors:** Jun-guang Wang, Ling-ling Ying, Pei-pei He

**Affiliations:** Department of Radiology, Ningbo Yinzhou No. 2 Hospital, Ningbo, Zhejiang, China

**Keywords:** prostate cancer, magnetic resonance imaging, lymph node metastasis, extraprostatic extension, biopsy positive rate

## Abstract

**Introduction:**

This study was conducted to evaluate any association between extraprostatic extension (EPE) grade with the risk of pelvic lymph node metastasis (PLNM) of prostate cancer (PCa).

**Methods:**

Magnetic resonance imaging data, as well as clinical and pathological data were collected for 317 patients undergoing radical prostatectomy (RP) along with pelvic lymph node dissection (PLND) at Ningbo Yinzhou No. 2 Hospital from January 2019 to January 2024. The collected magnetic resonance images were scored employing the EPE grade. The factors associated with PLNM were analyzed through Chi-square test and independent sample T-test. Independent risk factors associated with PLNM were identified through Multivariate analyses. The area under the curve (AUC) was calculated and the diagnostic performance of the model was assessed by analyzing the receiver operating characteristic (ROC) curve. The clinical net benefit of EPE grade, biopsy positive rate, and the combined model were examined using clinical decision curves.

**Results:**

Among 317 patients, 33had PLNM. Multifactor analysis demonstrated EPE grade and biopsy positive rate as independent risk factors for PLNM of PCa. The AUC of EPE grade and biopsy positive rate was, respectively, 0.879 and 0.877, and the diagnostic efficiency of PLNM between the two was not statistically significant (P > 0.05). However, when the two approaches were combined, the diagnostic efficiency improved significantly, and the AUC increased to 0.921 (P < 0.05). The analysis of the clinical decision curve revealed a significantly higher clinical net benefit of the combined model than that of the EPE grade and biopsy positive rate.

**Conclusions:**

The EPE grade and biopsy positive rate exhibit an independent correlation with PLNM of PCa. In addition, the combination of the two can significantly enhance the accuracy of predicting PLNM of PCa.

## 1 Introduction

Prostate cancer (PCa) is the most common cancer in men (1), and pelvic lymph node metastasis (PLNM) is an important factor associated with poor prognosis (2). Pelvic lymph node dissection (PLND) is the “gold standard” for PLNM diagnosis in PCa; however, the indication, scope, and patient benefit of PLND remain controversial (3). The European Association of Urology (EAU) guidelines recommend PLND when the risk of lymph node metastasis is predicted to be ≥5% using the Briganti nomogram (4), while the National Comprehensive Cancer Network (NCCN) recommend PLND when the risk of lymph node metastasis is predicted to be ≥2% using the MSKCC nomogram (5). Although sentinel lymph node biopsy has a diagnostic role, standard pelvic lymph node (SPLND) dissection can only remove 38% of suspicious lymph nodes (6). Multiple studies have shown that the number of lymph nodes obtained and the positive rate are positively correlated with the extent of PLND (7, 8). Heidenreich reported that the lymph node detection rate of extended pelvic lymph node dissection (EPLND) is approximately twice that of SPLND (9). Notably, EPLND significantly increases postoperative complications compared to SPLND (10).

Preoperative diagnosis of PLNM of PCa is crucial for accurate staging and determining optimal treatment. PLNM in PCa usually appear small on magnetic resonance imaging (MRI) (11), and if a threshold of 0.8-1.0cm in short diameter is employed as a criterion, the clinical stage may be underestimated due to low detection sensitivity (12). Ultra-small iron oxide particle MRI or choline positron emission tomography-computed tomography (PET-CT) has demonstrated high accuracy in detecting PLNM (13, 14), nonetheless, the executability of these techniques is low. Therefore, accurately predicting PLNM before radical prostatectomy (RP) of PCa remains the key and challenging aspect of clinical work.

Recent studies analyzing magnetic resonance images to quantitatively grade the likelihood of extraprostatic invasion in PCa, have shown that higher extraprostatic extension (EPE) grade is associated with higher tumor stage, Gleason score, or biochemical recurrence (15). According to these results, a higher EPE grade is associated with more aggressive PCa and a poorer prognosis. Consequently, EPE grade may also be associated with PLNM in PCa.

## 2 Materials and methods

### 2.1 Study population

**Figure 1:**
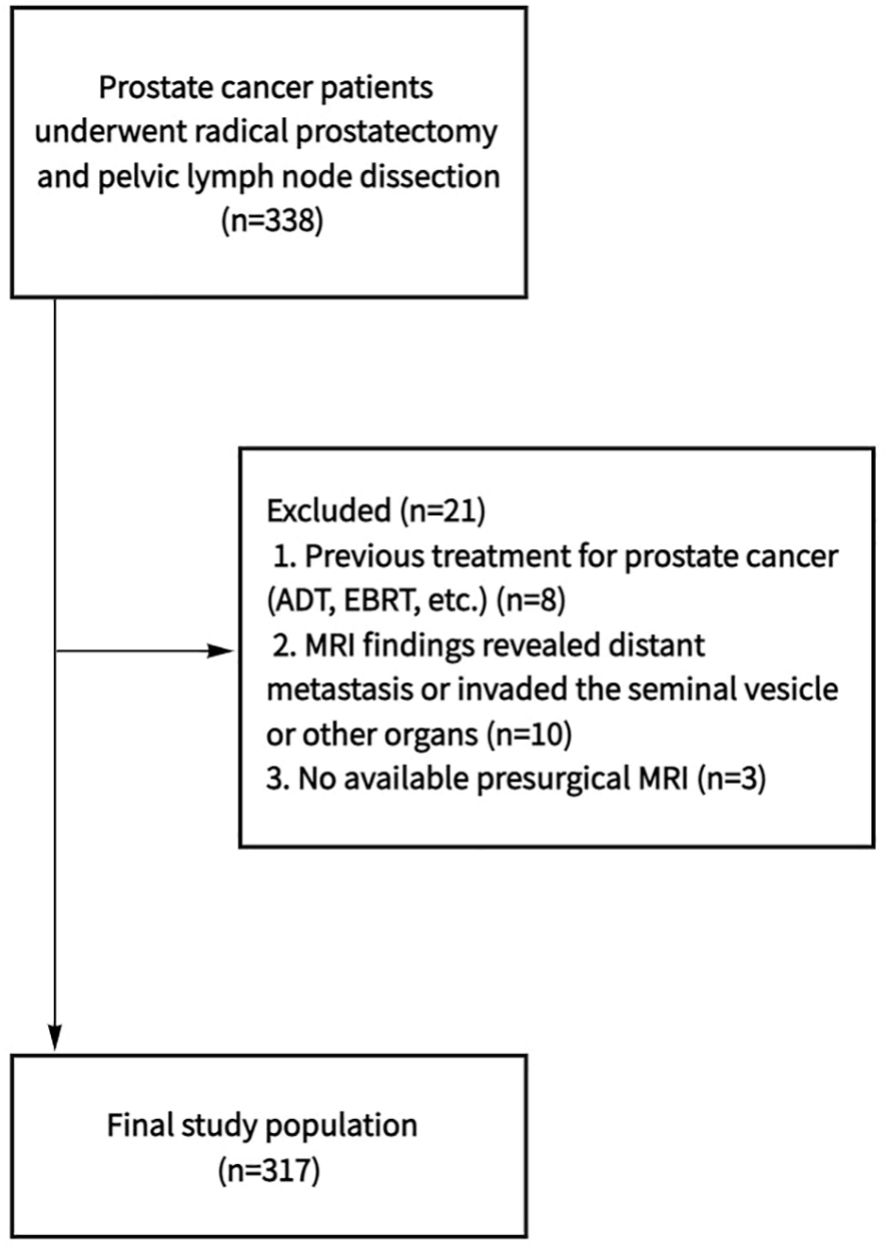
Flowchart shows determination of final study population. ADT, androgen deprivation therapy; EBRT, external beam radiation therapy; MRI, magnetic resonance imaging.

Clinical data of 338 patients who underwent radical prostatectomy (RP) and PLND at Ningbo Yinzhou No. 2 Hospital from January 2019 to January 2024 were collected. Inclusion criteria were: (1) clear magnetic resonance images, (2) less than 3 months of the interval between MRI examination and operation, and (3) complete clinicopathological data. Exclusion criteria: (1) neoadjuvant hormone therapy prior to surgery, (2) preoperative MRI images showed that the tumor had invaded the seminal vesicle or other organs, or imaging findings revealed distant metastasis. Finally, a total of 317 patients were included in the study (**Figure 1**).

### 2.2 Magnetic resonance imaging technology

Prostate MRI examination of all patients was performed 3 months prior to surgery using 1.5TMRI (GE SIGNA Voyager) scanner. The scan sequences included T1-weighted imaging (T1WI), dynamic enhanced T1WI, high-resolution T2-weighted imaging (T2WI), and diffusion-weighted imaging (DWI). The imaging scan area comprised the internal iliac artery bifurcation from the external iliac artery to the inguinal area.

### 2.3 Image analysis

**Figure 2:**
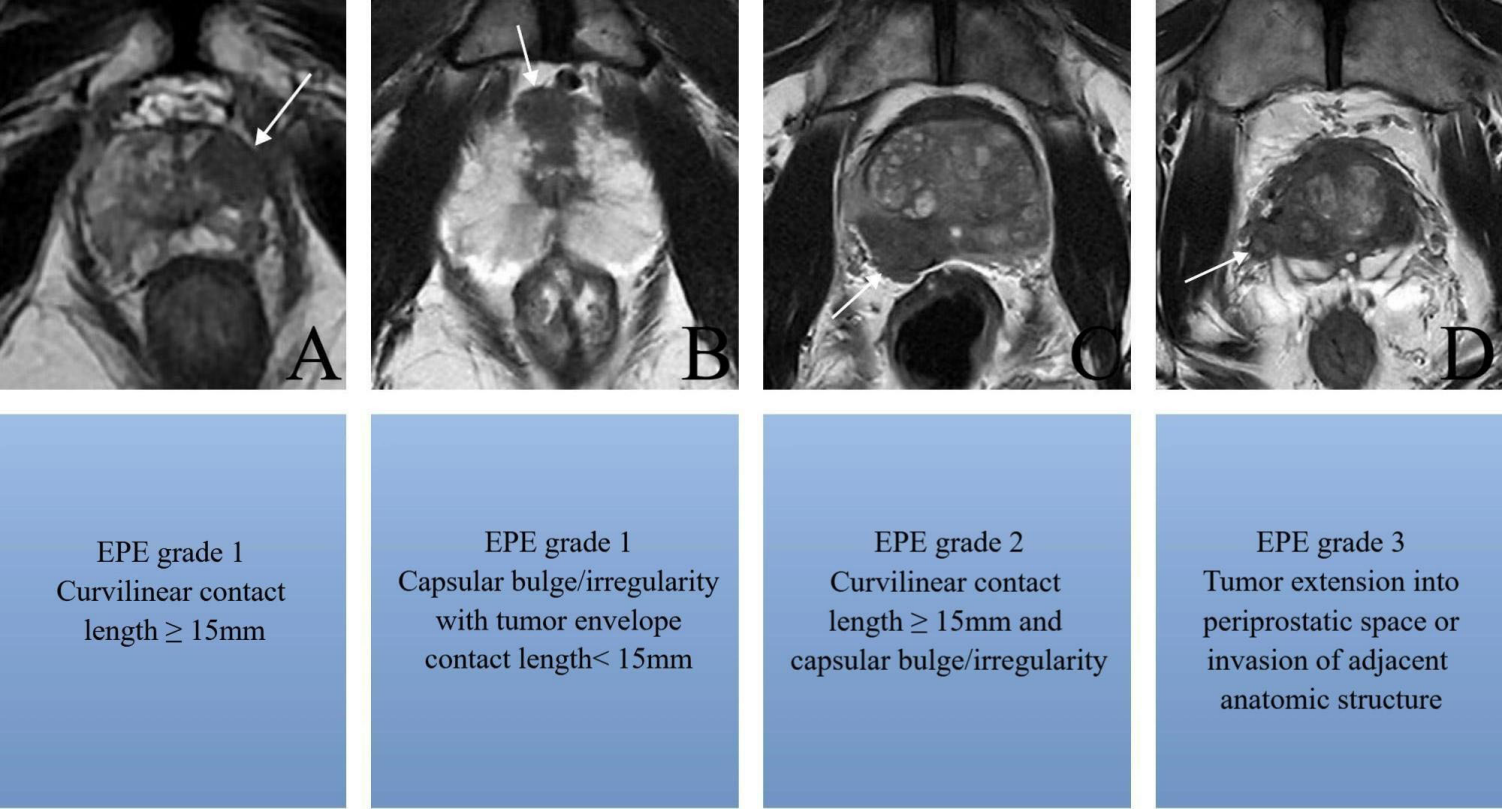
Axial T2-weighted MR images show **(A)** tumor envelope contact length ≥ 15mm without irregular envelope (arrow), **(B)** envelope swelling and irregular envelope with tumor envelope contact length< 15mm (arrow), **(C)** ≥15 mm contact length of the envelope with irregular and bulging envelope(arrow), **(D)** tumor extension into periprostatic space or invasion of adjacent anatomic structure in magnetic resonance images (arrow).

EPE grade were assessed by two experienced radiologists who were blinded to the PLND and pathology results during MRI review, as: grade 0: no suspicion of pathological extrinsic invasion, grade 1: envelope swelling or irregular envelope or tumor envelope contact length ≥ 15mm, grade 2: ≥15 mm contact length of the envelope, with irregular and bulging envelope, grade 3: tumor extension into periprostatic space or invasion of adjacent anatomic structure in magnetic resonance images (**Figure 2**). Drew a vertical line through the urethra as the center line to divide the prostate into left and right halves, and located the dominant tumor on the left and right sides respectively.

### 2.4 Clinical and pathological assessment

In the European Association of Urology Risk Group, patients of PCa categorized as moderate or high risk were treated with EPLND. This procedure includes the dissection of external and internal iliac lymph nodes, obturator lymph nodes, as well as common iliac lymph nodes, following the assessment by the surgeon on treatment effectiveness and possible complications. Prior to surgery, all patients underwent a standard transperineal prostate biopsy (12 needles); for suspected lesions on MRI images, an additional 1–3 needle biopsy was performed. Postoperative specimens were evaluated by two pathologists having more than 10 years of experience.

### 2.5 Variables

Clinical data included age, body mass index (BMI), prostate-specific antigen (PSA) level, prostate volume (PV), biopsy positive rate, biopsy grade group (GG) (Gleason score ≤6, 3 + 4, 4 + 3, 8, 9–10 corresponding to groups 1-5, respectively), highest percentage of cancer in positive cores, Clinical stage, and the EPE grade of PCa.

### 2.6 Statistical analysis

Statistical analysis was performed using SPSS(V17.0), Stata (V17.0), and MedCalc(V20.0). The Kappa test was used to evaluate the consistency between the two radiologists. Inter-group comparisons were carried out using the independent sample t-test or Chi-square test. Unless otherwise indicated, data were expressed as the median of the quartile range (IQRS). The independent risk factors for PLNM of PCa were determined through multivariate logistic regression analysis. The receiver operating characteristic (ROC) curves of the subjects corresponding to each independent risk factor and the combined model of independent risk factors were prepared. The area under the curves (AUC) was calculated, and the differences were compared employing the Delong test, the differences being statistically significant at P < 0.05. The decision analysis curve for each independent risk factor and the combined independent risk factor was drawn when predicting PLNM. Next, the clinical benefit of each independent risk factor as well as the combined independent risk factor in predicting PLNM were evaluated by comparing the relative position of each curve and the net profit rate corresponding to different risk thresholds.

## 3 Results

### 3.1 Descriptive statistics

This study included 317patients with PCa, 33of whom had PLNM, 8 (mean) Lymph Nodes were resected in PLNM+ group, 19 (58%). 8(24%) and 6 (18%) cases had 1, 2 and > 2 positive Lymph Node, while 16, 13and 4 cases had Lymph Node metastasis on the left (L), right (R), and bilaterally. The locations of dominant tumor with PLNM were as follows: 19 cases on the left side and 14 cases on the right side. Among the 19 cases with dominant tumor on the left side, 13 cases had left PLNM, 3 cases had right PLNM, and 3 case hade bilateral PLNM. Among the 14 cases with dominant tumor on the right side, 10 cases had right PLNM, 3 cases had left PLNM, and 1 case hade bilateral PLNM.

The median (IQR) age was 70 (66-74)years, the BMI was 22.9 (21.5-25.5) kg/m^2^, the PSA was 12.7 (6.1-19.6) ng/mL, the PV was31.8 (24.0-44.1) mL, the biopsy positive rate was 36 (20-58) %, and Highest percentage of cancer in positive cores was 48 (35-76)%. Biopsy GG 1, 2, 3, 4, and 5 corresponded to, respectively,30, 54, 82, 102, 49 cases. Clinical stage T1c, T2a, T2b, and T2c, respectively, corresponded to 121, 122, 46, and 28 cases. The EPE grades 0, 1, 2, and 3, respectively, corresponded to112, 104, 71, and 30cases (**Table 1**). The Kappa coefficient for observer consistency in EPE grade was 0.820, indicating a strong consistency.

**Table 1:** Patients’ characteristics (n=317).

Characteristic	Unit/Group	Median	IQR
Age	years	70	66-74
BMI	kg/m^2^	22.9	21.5-25.5
PSA	ng/ml	12.7	6.1-19.6
PV	ml	31.8	24.0-44.1
Biopsy positive rate	%	36	20-58
Biopsy GG n(%)	1	30	(15)
	2	54	(27)
	3	82	(41)
	4	102	(51)
	5	49	(25)
Highest percentage of cancer in positive cores	%	48	35-76
Clinical stage n(%)			
	T1c	121	(61)
	T2a	122	(61)
	T2b	46	(23)
	T2c	28	(14)
EPE grade n(%)	0	112	(56)
	1	104	(52)
	2	71	(36)
	3	30	(15)

BMI, body mass index; GG, grade group; IQR, interquartile range; PSA, prostate specific-antigen; PV, prostate volume.

### 3.2 Factors associated with pelvic lymph node metastasis

PSA levels were significantly higher in patients with positive PLNM than those in negative patients (37.7[19.7-64.7]*vs*.9.4 [6.0-16.1] ng/ml, p< 0.05). Patients with positive PLNM also had a higher biopsy-positive rate than that in patients with negative PLNM (75[60-88]*vs*.33[20-50]%, p< 0.05). Clinical stage, Biopsy GG and EPE grades also differed significantly between positive and negative patients with PLNM (p< 0.05) (**Table 2**). Multivariate analysis revealed that for PLNM of PCa, biopsy positive rate (p< 0.05) and EPE grade (p< 0.05) were independent risk factors (**Table 3**). When the EPE grade was greater than or equal to level 2, the Youden index was the largest. The sensitivity, specificity, negative predictive value, and positive predictive value for predicting PLNM were 90.9%, 75.7%, 98.6%, and 29.7%, respectively. The AUC of EPE grade for predicting lymph node metastasis was 0.879.

**Table 2:** Comparison of clinicopthological and mp-MRI factor.

Variables	PLNM +(n=33)	PLNM -(n=284)	P-value
Clinicopthological			
Age, years	71 (66-75)	70 (67-74)	0.984
BMI, g/m^2^	22.8 (20.1-24.9)	23.7 (21.6-25.6)	0.189
PSA, ng/ml	37.7 (19.7-64.7)	9.4 (6.0-16.1)	< 0.05
PV, ml	36.8 (27.6-45.2)	31.6 (23.9-43.8)	0.805
Biopsy GG, n(%)			< 0.05
1-2	1 (3.0)	83 (29.2)	
3-4	13 (39.4)	171 (60.2)	
5	19 (57.6)	30 (10.6)	
Biopsy positive rate, %	75 (60-88)	33 (20-50)	< 0.05
Highest percentage of cancer in positive cores	56(42-76)	48(35-71)	0.146
Clinical stage			< 0.05
T1c	10(30.3)	111(39.1)	
T2a	8(24.2)	114(40.1)	
T2b	8(24.2)	38(13.4)	
T2c	7(21.2)	21(7.4)	
MP-MRI			
EPE grade, n(%)			< 0.05
0-1	3 (9.1)	213 (75.0)	
2	13 (39.4)	58 (20.4)	
3	17 (51.5)	13 (4.6)	

Data are shown by median (IQR) unless otherwise indicated. BMI, body mass index; EPE, extraprostatic extension; GG, grade group; IQR, interquartile range; MRI, magnetic resonance imaging; PLNM, pelvic lymph node metastasis; PSA, prostate-specific antigen; PV, prostate volume.

**Table 3:** Multivariate analysis for predicting pelvic lymph node metastasis using clinical and MRI parameters (n=317).

Variables	N	OR	95%CI	P-value
PSA, ng/ml	317	1.006	0.989-1.022	0.517
Biopsy GG, n(%)				
1-2	83 (26.2)	ref.	ref.	ref.
3-4	185 (58.4)	1.171	0.120-11.462	0.892
5	49 (15.4)	3.314	0.319-34.436	0.316
Biopsy positive rate, %	317	17.265	1.467-203.250	< 0.05
Clinical stage, n(%)				
T1c	121(38.2)	ref.	ref.	ref.
T2a	122(38.5)	0.767	0.209-2.820	0.690
T2b	46(14.5)	0.425	0.099-1.834	0.252
T2c	28(8.8)	1.542	0.313-7.600	0.594
EPE grade, n(%)				
0-1	216 (68.1)	ref.	ref.	ref.
2	71 (22.4)	6.020	1.151-31.484	< 0.05
3	30 (9.5)	21.755	3.911-121.028	< 0.05

BMI, body mass index; CI, confidence interval; EPE, extraprostatic extension; GG, grade group; OR, odds ratio; PV, prostate volume; PSA, prostate-specific antigen; ref, reference.

### 3.3 Analysis of receiver operating characteristic curves of subjects with pelvic lymph node metastasis

**Figure 3:**
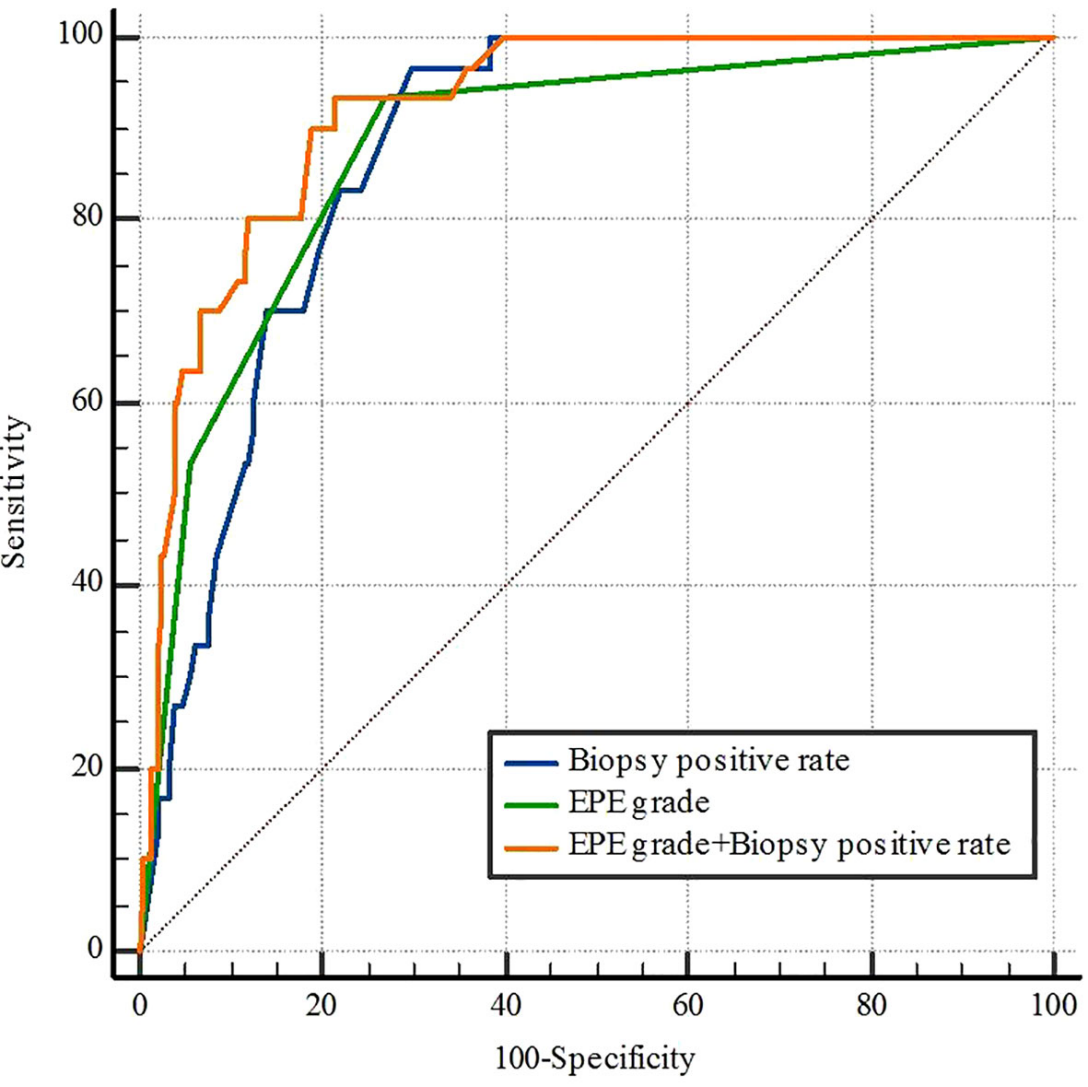
Receiver operating characteristic curves for biopsy positive rate, EPE grade, and EPE grade+ Biopsy positive rate of pelvic lymph node metastasis. EPE, extraprostatic extension.

The ROC curve analysis of subjects with PLNM revealed that the respective AUC of EPE grade and the biopsy positive rate were 0.879 (95%CI 0.838-0.912, p< 0.05) and 0.877 (95%CI 0.837-0.911) (p< 0.05) (**Table 4**) (**Figure 3**). The prediction value of EPE grade and biopsy positive rate for PLNM did not differ significantly (p > 0.05). Considering both EPE grade and biopsy positive rate (AUC = 0.921), the prediction performance of PLNM was improved significantly compared with any single index considered alone and the difference was statistically significant (p< 0.005).

**Table 4:** ROC analysis for pelvic lymph node metastasis.

Variables	AUC	95%CI	P-value
Biopsy positive rate	0.877	0.837-0.911	< 0.05
EPE grade	0.879	0.838-0.912	< 0.05
EPE grade+ Biopsy positive rate	0.921	0.887-0.948	< 0.05

AUC, area under curve; CI, confidence interval; EPE, extraprostatic extension; ROC, receiver operating characteristic.

### 3.4 The clinical decision curve of biopsy positive rate, extraprostatic extension grade, and combined model

**Figure 4:**
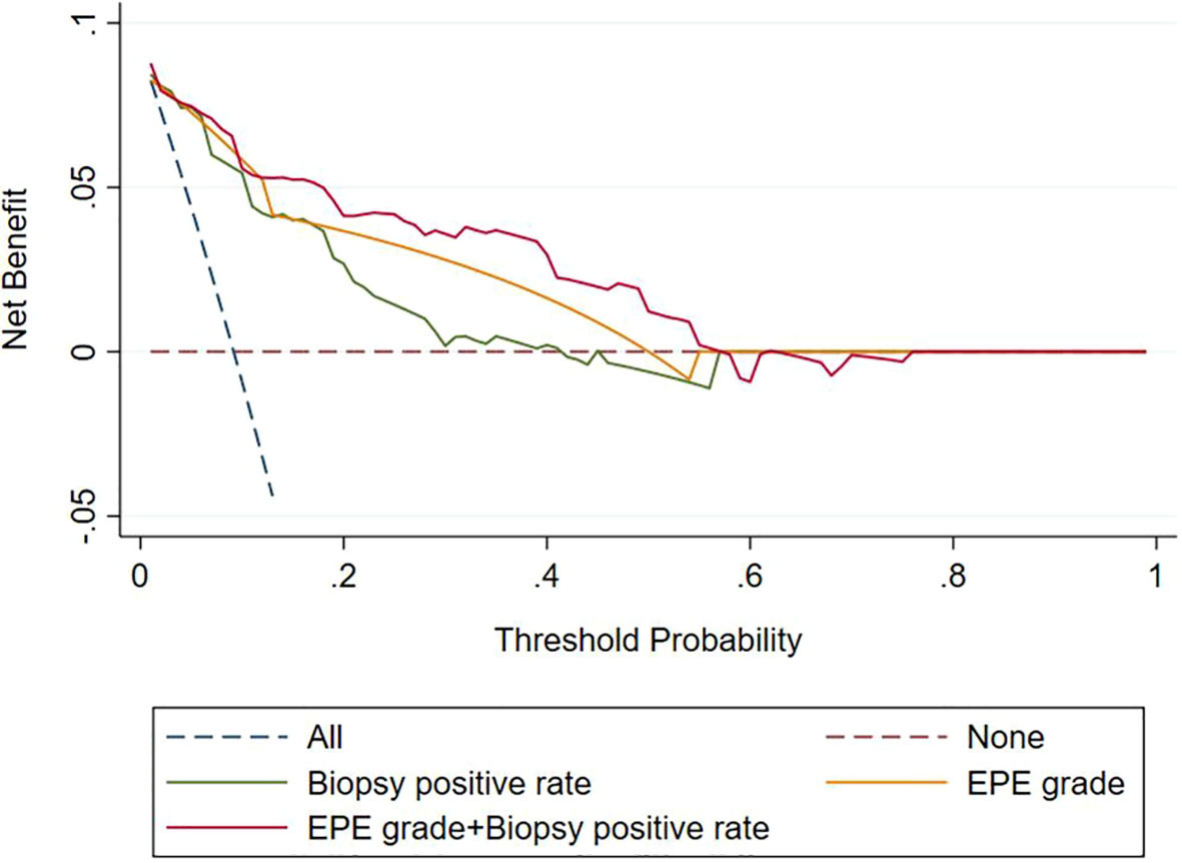
Decision curves of the biopsy positive rate, EPE grade, and EPE grade+ Biopsy positive rate for diagnosing pelvic lymph node metastasis. EPE, extraprostatic extension.

Analysis of the clinical decision curve, revealed that the biopsy positive rate, the EPE grade, and the combined model predicting PLNM were positioned at the upper right of the two extreme curves at different risk thresholds, indicating a higher net benefit to all participants. The combined model, at most risk thresholds, had a significantly higher net benefit than the biopsy positive rate and the EPE grade (**Figure 4**).

## 4 Discussion

We used preoperative MRI and clinicopathological data to assess the risk of PLNM in 317 patients with PCa undergoing RP and PLND and found EPE grade and biopsy positive rate as independent risk factors. A combination of these independent risk factors improved the accuracy of predicting PLNM in PCa.

At present, preoperative lymph node staging of PCa relies mainly on CT and MRI, but these imaging methods cannot identify PLNM. Hovels (16) found 39% and 42% sensitivity, respectively, of MRI and CT in predicting lymph node metastasis. PSMA PET-CT demonstrates low sensitivity and high specificity in predicting PLNM in PCa (17). The radiomics nomogram currently has good predictive efficacy for PLNM of PCa, but its reliability needs to be verified further (18, 19).

In this group, the metastasis rate of PLNM of PCa was 10.4%, and that of PLNM of EPE grades 0-I, 2, and 3 were, respectively, 1.4%, 18.3%, and 56.6%, with statistically significant differences between groups. The purpose of EPE grading is to stratify the risk of PCa extrinsic invasion. Studies on the efficacy of EPE grade have found the correlation of EPE grade with pathological Gleason score and clinical stage (20). The probability of lymph node metastasis also correlates highly with tumor stage and pathological Gleason score (21). Outside the prostate capsule, there is a dense lymphatic drainage network. As the tumor grows in size and breaks through the prostate capsule, the density of lymphatic vessels around the tumor increases, hastening the occurrence of pelvic lymph node metastasis of PCa (22). This may explain the study findings that the majority of patients with PLNM had high EPE grade on magnetic resonance images. Therefore, theoretically, the risk of PLNM increases significantly in patients with EPE grade 2 and 3. For such patients, surgical indications should be strictly adhered to, and the risk of PLNM must be fully explained to the patients and their families before surgery.

Clinicopathological factors PLNM of PCa with high accuracy, and PSA and Gleason scores are independent predictors of PLNM of PCa (23). Porcaro found a positive correlation between PSA level and PLNM (P = 0.012) (24), and Yiakoumos also reported PSA density as an important predictor of PLNM of PCa (25). One study based on the SEER database and the American Cancer Database demonstrated a very low risk of PLNM for PCa with a Gleason score of 6 or less and a significantly higher risk, with a Gleason score of 8-10 (26). The risk factors for PLNM of PCa include the number of positive needles and biopsy positive rate, and biopsy positive rate serves as an independent predictor (27). According to recent studies, peripheral monocyte count is also one of the best predictors of PLNM of PCa (28). In this study, the differences between positive and negative PLNM in PSA, biopsy positive rate, and biopsy GG were statistically significant, among which biopsy positive rate was an independent risk factor for PLNM of PCa.

A combination of MRI with clinicopathological indicators approach can enhance the accuracy of predicting PLNM of PCa. Multivariate models incorporating MRI tumor volume, tumor T stage, PSA, and biopsy GG demonstrated a high predictive value for PLNM in PCa (29). In this study, the AUC for predicting PLNM through EPE grade was 0.879, and the AUC for predicting PLNM increased to 0.921 upon employing the combination of EPE grade and the biopsy positive rate, with statistical significance. This study indicates that the combined biopsy positive rate and EPE grade has significant clinical value in predicting PLNM of PCa. To a certain extent, it can provide important reference for clinical treatment decisions regarding PCa. When the EPE grade is greater than or equal to level 2, it suggests that urologists should consider performing PLND for patients of PCa.

In this study, among the 33 patients of PCa in this group who had PLNM, 23 patients had PLNM on the same side as the main lesion of the tumor, 6 patients had PLNM on the contralateral side as the main lesion of the tumor, and 4 cases had bilateral PLNM simultaneously. This was quite similar to that reported by Weckermann (30). It indicates that unilateral lymph node dissection on the side where the main tumor is located alone has a relatively high risk of missing pelvic lymph node metastasis on the contralateral side.

The study also had some limitations. First, the sample size of PLNM+ cases (n=33) is small for multivariable regression, risking model over fitting, Small PLNM+ cohort inflates confidence intervals. Second, as a retrospective study, there are fixed limitations in data collection and a risk of selection bias. Thirdly, All patients came from the same center, resulting in a relatively small overall sample size, the evidence level of the research results is not high, and large-scale, multi-center, and prospective studies are needed for further verification. Lastly, MRI protocol (1.5T) may limit the assessment of EPE grade of PCa.

## 5 Conclusion

To conclude, there is an independent correlation between EPE grade and the biopsy positive rate with PLNM of PCa. A comprehensive evaluation of these factors is helpful in predicting the probability of PLNM of PCa before surgery, which will be helpful for urologists in deciding whether to perform PLND during RP.

## Data Availability

The original contributions presented in the study are included in the article/supplementary material. Further inquiries can be directed to the corresponding author.
